# Evaluation of machine learning algorithms for the prognosis of breast cancer from the Surveillance, Epidemiology, and End Results database

**DOI:** 10.1371/journal.pone.0280340

**Published:** 2023-01-26

**Authors:** Ruiyang Wu, Jing Luo, Hangyu Wan, Haiyan Zhang, Yewei Yuan, Huihua Hu, Jinyan Feng, Jing Wen, Yan Wang, Junyan Li, Qi Liang, Fengjiao Gan, Gang Zhang

**Affiliations:** Department of Breast and Thyroid Surgery, Sichuan Provincial Hospital for Women and Children (Affiliated Women and Children’s Hospital of Chengdu Medical College), Chengdu, China; International Centre for Genetic Engineering and Biotechnology, INDIA

## Abstract

**Introduction:**

Many researchers used machine learning (ML) to predict the prognosis of breast cancer (BC) patients and noticed that the ML model had good individualized prediction performance.

**Objective:**

The cohort study was intended to establish a reliable data analysis model by comparing the performance of 10 common ML algorithms and the the traditional American Joint Committee on Cancer (AJCC) stage, and used this model in Web application development to provide a good individualized prediction for others.

**Methods:**

This study included 63145 BC patients from the Surveillance, Epidemiology, and End Results database.

**Results:**

Through the performance of the 10 ML algorithms and 7th AJCC stage in the optimal test set, we found that in terms of 5-year overall survival, multivariate adaptive regression splines (MARS) had the highest area under the curve (AUC) value (0.831) and F1-score (0.608), and both sensitivity (0.737) and specificity (0.772) were relatively high. Besides, MARS showed a highest AUC value (0.831, 95%confidence interval: 0.820–0.842) in comparison to the other ML algorithms and 7th AJCC stage (all P < 0.05). MARS, the best performing model, was selected for web application development (https://w12251393.shinyapps.io/app2/).

**Conclusions:**

The comparative study of multiple forecasting models utilizing a large data noted that MARS based model achieved a much better performance compared to other ML algorithms and 7th AJCC stage in individualized estimation of survival of BC patients, which was very likely to be the next step towards precision medicine.

## Introduction

Breast cancer (BC) was the leading cancer in women, and BC alone accounted for 30% of newly diagnosed cancers in American women in 2019 [1]. Assessing the prognosis of BC patients could significantly affect the choice of the best treatment plan. For example, for patients with a poor prognosis, they may choose a more aggressive treatment. The most important predicting tool, the one that remained in worldwide use today, was the American Joint Committee on Cancer (AJCC) staging system [2]. There, however, were several evidence here that the traditional AJCC staging system could not accurately assess the prognosis of BC patients [3–5]. Many complex factors affected the prognosis of cancer patients, so survival prediction for cancer patients was a challenging task. In this context, modern oncology has witnessed the growing interest in digital technology, and the integration of digital technology and large medical data has brought new hope for personalized medicine.

Machine learning (ML) is a branch of artificial intelligence that employed a variety of statistical, probabilistic and optimization techniques that allowed computers to “learn” from past examples and to detect hard-to-discern patterns from large, noisy or complex data sets [6]. Many articles used ML to predict the prognosis of many cancer patients, including BC, lung cancer, and liver cancer, and noticed that the ML model had good individualized prediction performance [7–29]. For example, Kalafi et al [12] presented that multilayer perceptron produced desirable prediction accuracy for predicting the prognosis of BC patients. Tahmassebi et al [13] proposed that extreme gradient boosting with multiparametric magnetic resonance imaging achieved stable performance for the early prediction of pathological complete response to neoadjuvant chemotherapy and of survival outcomes in BC patients. Poirion et al [14] introduced a novel ensemble framework of deep-learning and machine-learning approaches that robustly predicted BC patient survival subtypes using multi-omics data. A retrospective study on predicting 10-year survival after breast cancer surgery revealed that all performance indices for the deep neural network model were significantly higher than in the other forecasting models [16]. Liu et al [27] proposed a gradient boosting algorithm by optimizing survival analysis of XGBoost framework for ties to predict the disease progression of breast cancer. ML, therefore, was very likely to be the next step towards precision medicine.

Since ML models were susceptible to factors such as data sources, input variables, and software, several articles using ML to predict the prognosis of BC patients were controversial [7–9, 12–16, 20, 25–28]. Lotfnezhad Afshar et al [9] believed that support vector machine (SVM) model outperformed other models in the predicting the survival rate of BC patients. Moreover, Delen et al [30] indicated that the decision tree (DT) was the best predictor. Furthermore, a retrospective study proposed that random forest (RF) model showed a better diagnostic performance for predicting recurrence than did the five other machine learning classifiers [25]. Additionally, it should be mentioned that although some researchers claimed that these ML techniques could effectively predict the prognosis of patient, few people were actually used in clinical practice. This study was intended to establish a reliable data analysis model by comparing the performance of 10 common ML algorithms and the traditional AJCC staging system based on a national database, and used this model in Web application development to provide a good individualized prediction for others.

## Materials and methods

### Database and samples

The Surveillance, Epidemiology, and End Results (SEER) Program of the National Cancer Institute was an authoritative source of information on cancer incidence and survival in the United States and covered approximately 48.0% of the United States population [31]. Although the SEER database had some limitations, such as lack of certain data (such as postoperative complications, surgical margin, recurrence, etc.), its multi-center and large sample characteristics were suitable for building a ML model for the general population.

**Fig 1:**
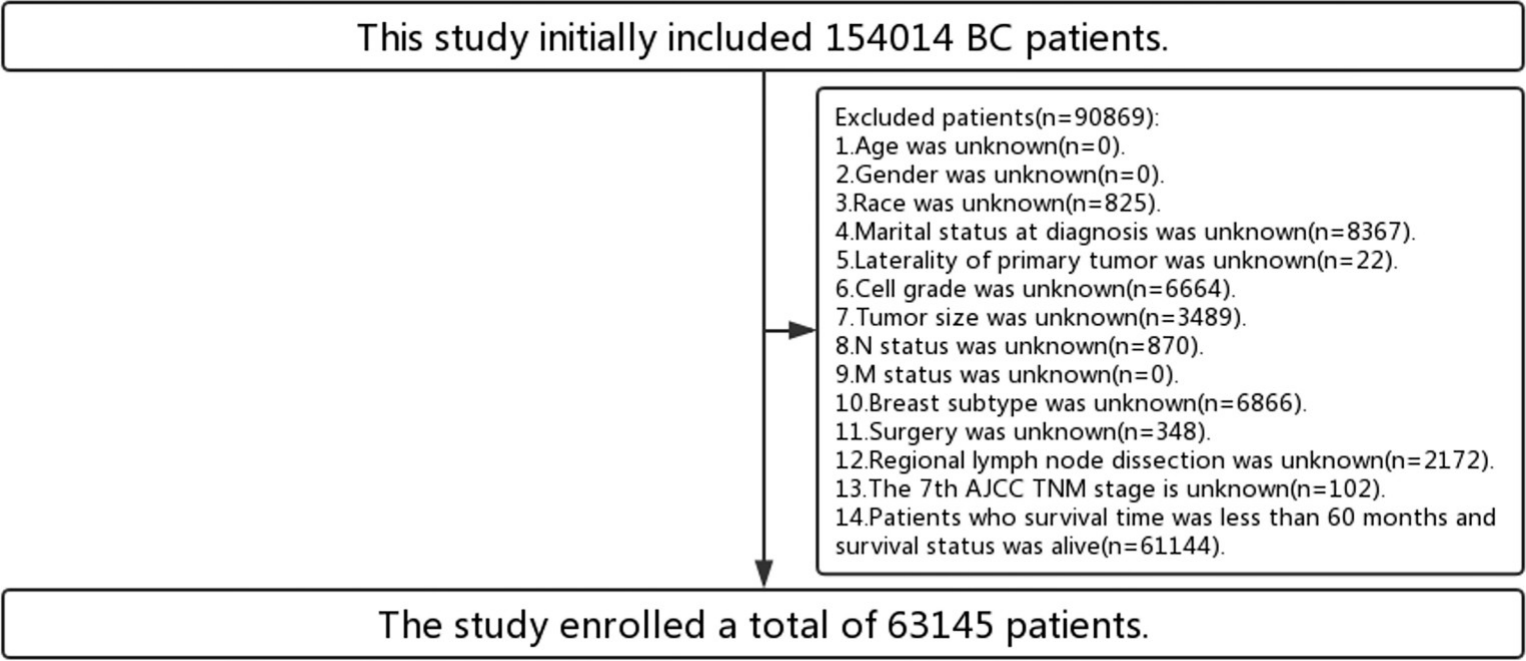
The inclusion and exclusion process of this study.

The data of BC patients for this study was acquired from the SEER database, and it included 154014 patients based on the fact that year of diagnosis was from 2010 to 2014, primary tumor site was coded as C50.0 to C50.6 (including C50.0-Nipple, C50.1-Central portion of breast, C50.2-Upper-inner quadrant of breast, C50.3-Lower-inner quadrant of breast, C50.4-Upper-outer quadrant of breast, C50.5-Lower-outer quadrant of breast, C50.6-Axillary tail of breast), behavior recode for analysis was malignant, and diagnostic confirmation was positive histology. The study enrolled a total of 63145 patients by excluding patients with missing data and patients who survival time was less than 60 months and survival status was alive (**Fig 1**). The final endpoints of this study were the 5-year overall survival (OS) rate, so we excluded patients who survival time was less than 60 months and survival status was alive.

### Statistical analysis

**Fig 2:**
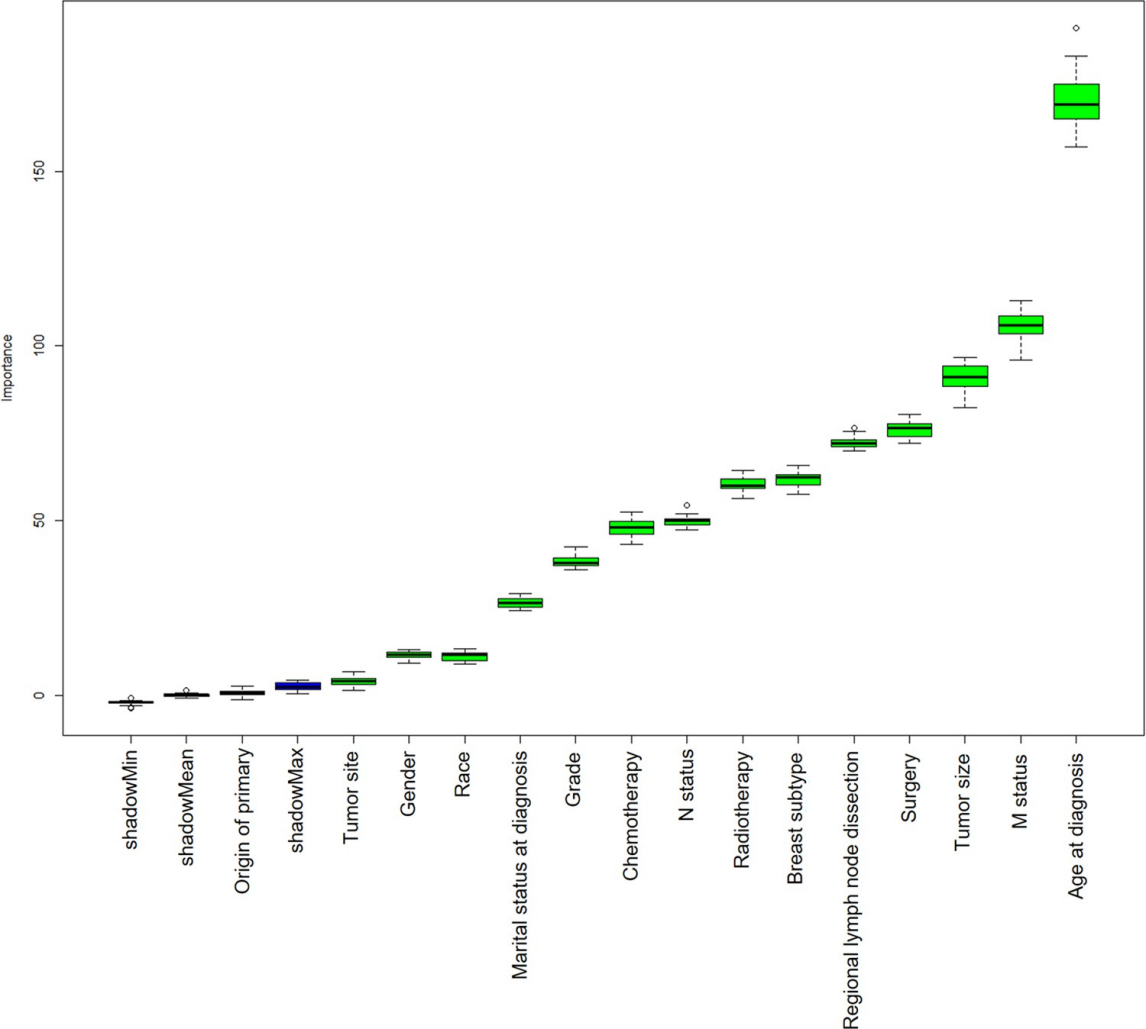
Feature selection for the 10 ML models.

Categorical variables were presented as frequency and percentage, and continuous variables were presented as mean (x) and standard deviation (s). This study could only obtain the staging information of 7th AJCC due to the SEER database. For ML models, we extracted 15 factors that may affect the prognosis of patients from the SEER database based on professional knowledge, including age at diagnosis, gender, race, marital status at diagnosis, tumor site, origin of primary, grade, tumor size, N status, M status, breast subtype, surgery, regional lymph node dissection, chemotherapy, and radiotherapy (see the results for details). We used the Boruta package [32] in the R software for feature selection and found that 14 attributes other than origin of primary were confirmed important (**Fig 2**). It found relevant features by comparing original attributes’ importance with importance achievable at random, estimated using their permuted copies (shadows). We, therefore, included these 14 covariates in the 10 ML models.

In order to reduce the over-fitting of the model and ensure the robustness of the model, we used the 9-fold cross-validation method to select the test set with the centered area under the curve (AUC) value as the optimal test set by the caret package [33]. Using DeLong test to compare the AUC values of different ML algorithms and 7th AJCC stage in the optimal test set, the best performing model was selected for web application development by shiny package [34] and shinydashboard package [35]. We utilized the accuracy, F1-score, sensitivity, specificity, and AUC to evaluate the performance of models for each prediction case.

Common ML algorithms, such as naive bayes (NB), linear discriminant analysis (LDA), quadratic discriminant analysis (QDA), k-nearest neighbor (KNN), SVM, classification and regression trees (CART), RF, multivariate adaptive regression splines (MARS), logistic regression (LR), and extreme gradient boosting (XGBoost), were selected in this study. For each of these functions, we used the relevant package’s default parameters, see below for details.

NB computed the conditional a-posterior probabilities of a categorical class variable given independent predictor variables using the Bayes rule. Although it assumed that the presence/absence of a characteristic describing a certain class was unrelated to the presence/absence of any other characteristic, which was not true for the majority of classification tasks, NB have been successful in complex practical applications [36]. The analysis of NB in this study was realized by the e1071 package in R [37].

Discriminant analysis was to summarize the rules from the various classifications of the known samples to determine the type of the new sample, including LDA and QDA. The difference between the two was that LDA was based on the assumption that the variables were multivariate normally distributed in each group with different mean vectors and identical covariance matrices, and while the equality of covariance assumption was not required in QDA, so this was the basic reason that LDA was a much less flexible classifier than QDA [38]. The discriminant analysis in this study was realized by the MASS package in R [39].

KNN, a nonparametric clustering algorithm, was used for data classification and regression [40], which predicted the information of the test sample through the information of the k training samples closest to the test sample in the train set. The analysis of KNN in this study was realized by the kknn package in R [41].

The basic idea of SVM was to get the separation hyperplane that could divide the data set correctly and had the largest geometric interval, and used the hyperplane to reasonably divide the data. The analysis of SVM in this study was realized by the kernlab package in R [42].

The CART model, a machine-learning and data-mining recursive algorithm, was used to identify groups of patients with a homogeneous risk of death and investigate the hierarchical association between variables and survival [43]. No pruning was done on the model. The analysis of CART in this study was realized by the rpart package in R [44].

RF was an ensemble learning method based on decision tree. In this study, the min number of trees grown obtained by randomForest package [45] in the train set was 441, and it was verified in the test set.

MARS was a non-parametric modelling method that extends the linear model, incorporating nonlinearities and interactions between variables. It was a flexible tool that automated the construction of predictive models [46]. The analysis of MARS in this study was realized by the earth package in R [47].

LR was one of the most important models in generalize linear model (GLM). It was mainly used to study the relationship between two-element categorical response variables ("success" and "failure" are represented by 1 and 0 respectively) and many covariates, and to establish corresponding models and make predictions.

The algorithm of XGBoost was a gradient-boosting decision tree that can be used for both classification and regression problems [48]. The greedy method optimized the maximal gain of the objective function during the construction of each tree layer [49]. The analysis of XGBoost in this study was realized by the xgboost package [50].

To further analyze the best performing model, we needed to evaluate the variable importance in this model. According to the Results section, MARS was the best performing model. We used three criteria to estimate the variable importance of the model through the evimp functions that came with the earth package [51]: (i)The nsubsets criterion counted the number of model subsets that included the variable. Variables that were included in more subsets were considered more important. (ii)The residual sum-of-squares (RSS) criterion first calculated the decrease in the RSS for each subset relative to the previous subset during earth’s backward pass. Then for each variable it summed these decreases over all subsets that included the variable. Finally, for ease of interpretation the summed decreases were scaled so the largest summed decrease was 100. Variables which caused larger net decreases in the RSS were considered more important. (iii)The generalized cross validation (GCV) criterion was the same, but used the GCV instead of the RSS. Adding the variable had a deleterious effect on the model, as measured in terms of its estimated predictive power on unseen data. Statistical analysis were conducted using R software 4.1.0.

Ethics statement was not required for this study, because this observational study used de-identified and publicly available data from SEER. This study was conducted in accordance with the Declaration of Helsinki. In addition, Data-Use Agreements for the 1975–2017 SEER Research Data File and SEER Radiation Therapy and Chemotherapy Information were signed and the database can be accessed.

## Results

### Baseline characteristics

Descriptive characteristics of 63145 BC patients were summarized in **Table 1**. The average age of the patients was 62.6 ± 13.8 years, and 81.1% of the patients were the white. As of the follow-up time (November 2019), a total of 15734 patients died, and the 5-year OS was 75.1%.

**Table 1:** Descriptive characteristics of 63145 BC patients.

Factors	`x ± s / N (%)	Factors
Age at diagnosis (years)		62.6 ± 13.8
Gender	female	62565(99.1)
	male	580(0.9)
Race	white	51216(81.1)
	black	6949(11.0)
	other^a^	4980(7.9)
Marital status at diagnosis	single (never married)/unmarried or domestic Partner	9240(14.6)
	married (including common law)	34971(55.4)
	divorced/widowed/separated	18934(30.0)
Tumor site	nipple	405(0.6)
	central portion	5361(8.5)
	upper-outer quadrant	32429(51.4)
	lower-outer quadrant	7221(11.4)
	lower-inner quadrant	5628(8.9)
	upper-inner quadrant	11574(18.3)
	axillary tail	527(0.8)
Origin of primary	left	32271(51.1)
	right	30874(48.9)
Grade	well differentiated	14431(22.9)
	moderately differentiated	27137(43.0)
	poorly differentiated	21314(33.8)
	undifferentiated	263(0.4)
Tumor size (mm)		22.4 ± 21.4
N status	positive	19801(31.4)
	negative	43344(68.6)
M status	positive	2469(3.9)
	negative	60676(96.1)
Breast subtype	luminal A	46290(73.3)
	luminal B	5891(9.3)
	HER2 enriched	2562(4.1)
	triple negative	8402(13.3)
Surgery	no	2986(4.7)
	radical mastectomy	186(0.3)
	extended radical mastectomy	11(0.0)
	modified radical mastectomy	11281(17.9)
	total mastectomy	14165(22.4)
	breast-conserving surgery	34516(54.7)
Regional lymph node dissection	no	6643(10.5)
	yes	56502(89.5)
Chemotherapy	no/unknown	37772(59.8)
	yes	25373(40.2)
Radiotherapy	no/unknown	30832(48.8)
	yes	32313(51.2)
7th AJCC stage	0 stage	11(0.0)
	IA stage	30801(48.8)
	IB stage	1520(2.4)
	IIA stage	14323(22.5)
	IIB stage	6926(11.0)
	IIIA stage	4097(6.5)
	IIIB stage	1178(1.9)
	IIIC stage	1918(3.0)
	IV stage	2462(3.9)

^a^: The other comprises American Indian/Alaska Native, Asian/Pacific Islander.

### Machine learning algorithms and 7th AJCC stage

**Fig 3:**
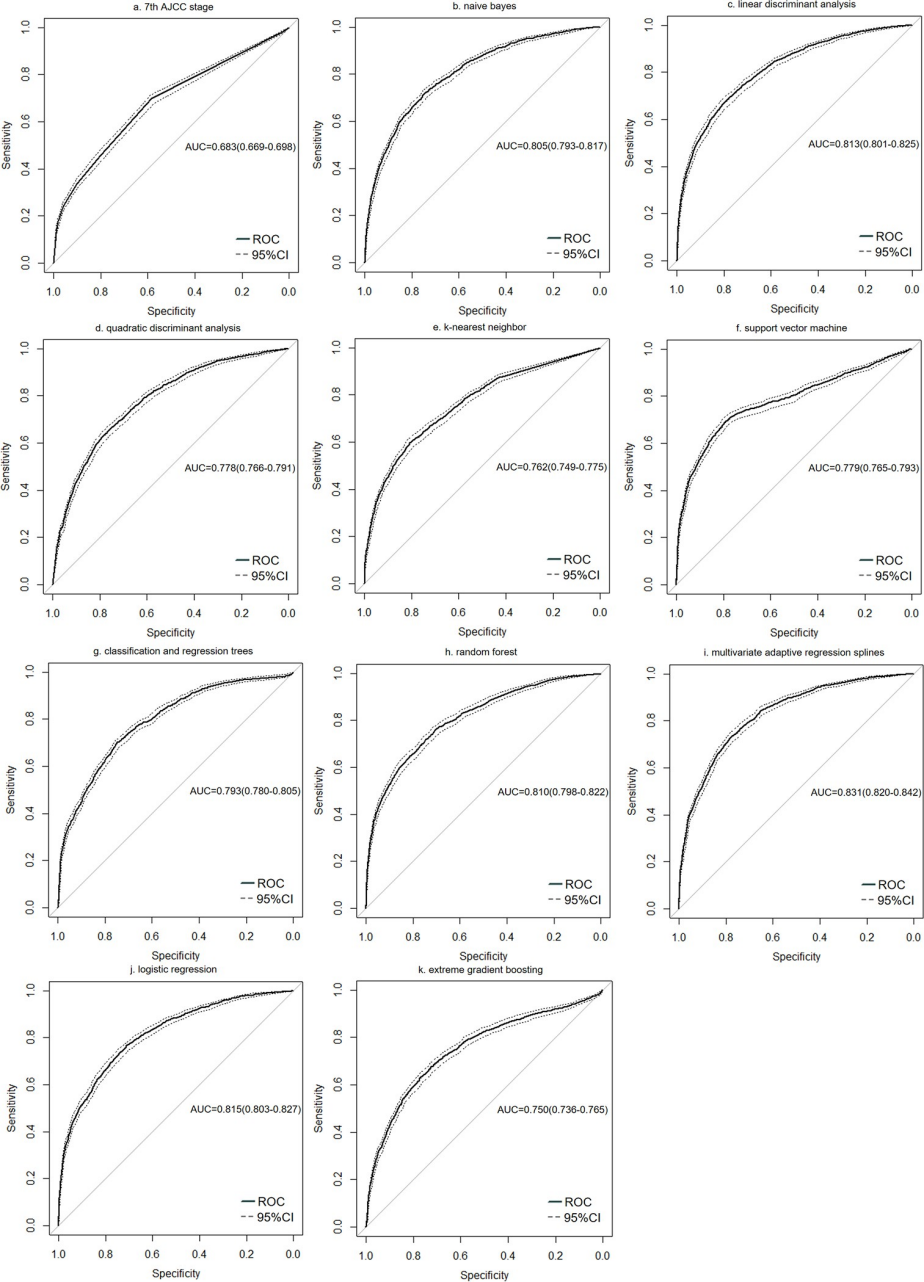
The ROC curves of 10 ML algorithms and 7th AJCC stage on 5-year OS in the test set.

Through the performance of the 10 ML algorithms and 7th AJCC stage in the test set (**Tables 2, 3** and **Fig 3**), the results showed that in terms of 5-year OS, LDA had the highest accuracy (0.771), higher specificity (0.806) and higher AUC value (0.813), but lower sensitivity (0.665). MARS had the highest AUC value (0.831) and F1-score (0.608), and both sensitivity (0.737) and specificity (0.772) were relatively high. Besides, MARS showed a highest AUC value (0.831, 95%confidence interval: 0.820–0.842) in comparison to the other ML algorithms and 7th AJCC stage (all P < 0.05, **Table 3**). The best forecasting ability among these models was MARS. The algorithms with the highest sensitivity was RF (0.763). KNN showed the highest specificity (0.807) and the lowest sensitivity (0.596).

**Table 2:** The accuracy, F1-score, sensitivity, specificity, and AUC value of 10 ML algorithms and 7th AJCC stage in the test set.

Model	Accuracy	F1-score	Sensitivity	Specificity	AUC	95%CI
7th AJCC	0.612	0.473	0.699	0.583	0.683	0.669–0.698
NB	0.741	0.579	0.716	0.749	0.805	0.793–0.817
LDA	0.771	0.591	0.665	0.806	0.813	0.801–0.825
QDA	0.750	0.555	0.625	0.791	0.778	0.766–0.791
KNN	0.754	0.547	0.596	0.807	0.762	0.749–0.775
SVM	0.766	0.596	0.692	0.790	0.779	0.765–0.793
CART	0.736	0.570	0.702	0.748	0.793	0.780–0.805
RF	0.717	0.573	0.763	0.701	0.810	0.798–0.822
MARS	0.764	0.608	0.737	0.772	0.831	0.820–0.842
LR	0.739	0.585	0.737	0.740	0.815	0.803–0.827
XGB	0.713	0.539	0.677	0.724	0.750	0.736–0.765

**Table 3:** The comparison of AUC values for 10 ML algorithms and 7th AJCC stage in the test set.

Model	7th AJCC	NB	LDA	QDA	KNN	SVM	CART	RF	MARS	LR
	P	P	P	P	P	P	P	P	P	P
7th AJCC	ref	-	-	-	-	-	-	-	-	-
NB	<0.001	ref	-	-	-	-	-	-	-	-
LDA	<0.001	0.323	ref	-	-	-	-	-	-	-
QDA	<0.001	0.002	<0.001	ref	-	-	-	-	-	-
KNN	<0.001	<0.001	<0.001	0.083	ref	-	-	-	-	-
SVM	<0.001	0.006	<0.001	0.943	0.089	ref	-	-	-	-
CART	<0.001	0.163	0.018	0.010	<0.001	0.151	ref	-	-	-
RF	<0.001	0.527	0.728	<0.001	<0.001	<0.001	<0.001	ref	-	-
MARS	<0.001	0.001	0.027	<0.001	<0.001	<0.001	<0.001	0.011	ref	-
LR	<0.001	0.237	0.847	<0.001	<0.001	<0.001	0.010	0.589	0.043	ref
XGB	<0.001	<0.001	<0.001	0.004	0.248	0.005	<0.001	<0.001	<0.001	<0.001

### Evaluating variable importance in the MARS model

By evaluating variable importance in the MARS model, we noticed that age at diagnosis was considered the most important variable, followed by tumor size, M status, regional lymph node dissection, N status, Breast subtype, and so on (**Table 4**).

**Table 4:** The evaluation of variable importance in the MARS model.

Variable	nsubsets	GCV(%)	RSS(%)
Age at diagnosis	21	100.0	100.0
Tumor size	20	79.7	79.8
M status	19	62.5	62.7
Regional lymph node dissection	18	48.3	48.6
N status	17	42.2	42.5
Breast subtype	17	42.2	42.5
Radiotherapy	15	32.5	33.0
Grade	14	28.1	28.6
Marital status at diagnosis	12	21.5	22.1
Race	11	18.7	19.3
Chemotherapy	8	14.1	14.7
Surgery	6	10.6	11.1
Gender	0	0.0	0.0
Tumor site	0	0.0	0.0
Origin of primary	0	0.0	0.0

### Web application development

**Fig 4:**
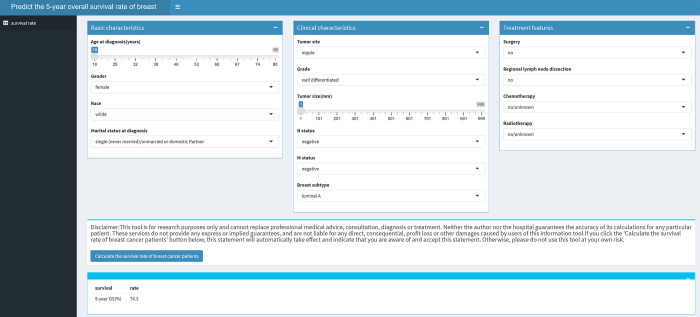
An example showing web function (https://w12251393.shinyapps.io/app2/).

We selected MARS model for web application development for other users to use for free based on the AUC value (https://w12251393.shinyapps.io/app2/). This web application could automatic calculate the 5-year OS according to the characteristics of the patient selected by the user. **Fig 4** was an example showing web function.

## Discussion

ML models could be defined as a process of designing a model and improving its performance through empirical learning. It were a field of artificial intelligence and an active research field in different scientific fields. Complex ML models could pick up on subtler patterns in input data and thus could be more effective predictors [52]. ML, therefore, was very likely to be the next step towards precision medicine.

In our research, ROC curve analysis showed that the AUC value of the 7th AJCC stage was 0.683 (95%CI: 0.669–0.698, **Fig 3A**), which was in the range of 0.620 to 0.728 previously studied [53–55]. The research conducted DeLong test on more than 60000 BC patients and found that the 10 ML algorithms had a better role in predicting the 5-year OS compared to 7th AJCC stage (all P < 0.001, **Table 3**). In the meantime, the 7th AJCC stage showed the lowest accuracy (0.612) and F1-score (0.473). There, for all we know, were no relevant researches comparing the predictive ability of AJCC stage and ML models for BC patients. We could only obtain the staging information of 7th AJCC due to the guarantee of a 5-year follow-up period and the limitation of the SEER database, while some researchers believed that the latest 8th AJCC stage still could not accurately stratify the prognosis for BC patients [4, 56]. For example, a study about the comparison of the prognostic accuracy of the 8th AJCC prognostic staging system to the 7th staging system using data from over 168000 BC patients confirmed the enhanced value of the 8th AJCC, while the latter still needed further improvement [56]. Furthermore, though several research results noticed that the AUC value of AJCC stage had risen from the 0.620–0.728 range of the 7th edition to the 0.670–0.773 range of the 8th edition [53–55], there was still a certain distance from the AUC value of MARS model in this study (AUC: 0.831, 95%CI: 0.820–0.842).

The result noted that MARS had the best performance among the 10 ML algorithms and 7th AJCC stage in predicting the 5-year OS of BC patients (**Tables 2** and **3**). MARS was a non-parametric modelling method that extended the linear model, incorporating nonlinearities and interactions between variables. It was a flexible tool that automated the construction of predictive models [46]. There was currently no study using the MARS model to predict the prognosis for BC patients, to the best of our knowledge. Several articles using different ML models to predict the prognosis of BC patients aroused controversy [7–9, 14–16, 20, 25–28, 57]. Firstly, Kate et al [7] believed that NB was better than DT and LR through the research on more than 160000 BC patients. Moreover, a meta-analysis of 11 articles about ML algorithms for BC risk calculation confirmed that the SVM algorithm was able to calculate breast cancer risk with better accuracy value than other ML algorithms [57], but this article did not include the MARS algorithm. Since ML models were susceptible to factors such as data sources, input variables, and software, and the number of ML algorithms compared by many studies was different, so it was difficult to directly compare with the results of other studies.

To our surprise, this study noted that age at diagnosis was considered the most important variable, even ahead of distant metastasis. While several findings proposed that age was as an independent prognostic factor for BC [58–60], by it has been well documented that metastases was the main cause of death for patients with breast cancer [61]. Estimating predictor importance, as everyone knows, was in general a tricky and even controversial problem. The evimp function was useful in practice for MARS model but the following issues could make it misleading [51]. For example, collinear (or otherwise related) variables could mask each other’s importance, just as in linear models; this meaned that if two predictors were closely related, the earth model building algorithm would somewhat arbitrarily choose one over the other [51]. The chosen predictor would incorrectly appear more important [51]. So estimates of predictor importance could be unreliable because they could vary with different training data.

Nonetheless, there were some advantages of this research. Firstly, the data of this study came from the SEER database, which was one of the most representative large tumor databases in North America. Moreover, we compared the accuracy, F1-score, sensitivity, specificity, and AUC values of 10 ML algorithms in detail and reported the P values of the AUC values, while some other studies used less than 5 ML algorithms and rarely reported their P values [7–9, 14]. More significantly, we used the selected ML model in Web application development to provide a good individualized prediction for others online.

This study also had several limitations. Firstly, the SEER database lacked some data effected on the prognosis of patients, such as postoperative complications, surgical margin, and recurrence. Secondly, the models in this study were all trained and tested on different parts of the same data set. Ideally, the model would be trained on one data set and validated on another separately studied data set. This external verification could prove the universality of the model. We could not use another external data set for external verification, so we had to divide the data set into train set and test set. Although this research used the 9-fold cross-validation method to reduce the over-fitting of the model and ensure the robustness of the model, whether these ML models could be well generalized to new data sets required further research. Thirdly, compared with traditional statistical models, ML algorithms had black box characteristics. Interpretation and understanding of the ML model was a key issue. Fourthly, since the sample size of this study exceeded 60000 and computation time required to use deep learning was too long, this study did not test deep models, which may affect the results of this study. Future work could be carried out to get a more accurate predictive model by including more ML algorithms, such as deep learning modes.

## Conclusions

The comparative study of multiple forecasting models utilizing a large data noted that MARS based model achieved a much better performance compared to other ML algorithms and 7th AJCC stage in individualized estimation of survival of BC patients, which was very likely to be the next step towards precision medicine.

## Supporting information

S1 Checklist PLOS ONE clinical studies checklist. (DOCX)

S2 Checklist STROBE statement—checklist of items that should be included in reports of observational studies. (DOCX)

## References

[1] Siegel RL, Miller KD, Jemal A. Cancer statistics, 2019. CA Cancer J Clin. 2019;69(1):7–34. doi: 10.3322/caac.21551 30620402

[2] Edge SB, Hortobagyi GN, Giuliano AE. New and important changes in breast cancer TNM: incorporation of biologic factors into staging. Expert Rev Anticancer Ther. 2019;19(4):309–318. doi: 10.1080/14737140.2019.1582335 30759347

[3] Chen S, Liu Y, Yang J, Liu Q, You H, Dong Y, et al. Development and Validation of a Nomogram for Predicting Survival in Male Patients With Breast Cancer. Front Oncol. 2019;9:361. doi: 10.3389/fonc.2019.00361 31139562 PMC6527749

[4] Yang Y, Wang Y, Deng H, Tan C, Li Q, He Z, et al. Development and validation of nomograms predicting survival in Chinese patients with triple negative breast cancer. BMC Cancer. 2019;19(1):541. doi: 10.1186/s12885-019-5703-4 31170946 PMC6555047

[5] Shi H, Wang XH, Gu JW, Guo GL. Development and Validation of Nomograms for Predicting the Prognosis of Triple-Negative Breast Cancer Patients Based on 379 Chinese Patients. Cancer Manag Res. 2019;11:10827–10839. doi: 10.2147/CMAR.S234926 31920392 PMC6941602

[6] Cruz JA, Wishart DS. Applications of machine learning in cancer prediction and prognosis. Cancer Inform. 2007;2:59–77. 19458758 PMC2675494

[7] Kate RJ, Nadig R. Stage-specific predictive models for breast cancer survivability. Int J Med Inform. 2017;97:304–311. doi: 10.1016/j.ijmedinf.2016.11.001 27919388

[8] Shukla N, Hagenbuchner M, Win KT, Yang J. Breast cancer data analysis for survivability studies and prediction. Comput Methods Programs Biomed. 2018;155:199–208. doi: 10.1016/j.cmpb.2017.12.011 29512500

[9] Lotfnezhad Afshar H, Ahmadi M, Roudbari M, Sadoughi F. Prediction of breast cancer survival through knowledge discovery in databases. Glob J Health Sci. 2015;7(4):392–8. doi: 10.5539/gjhs.v7n4p392 25946945 PMC4802184

[10] Lynch CM, Abdollahi B, Fuqua JD, de Carlo AR, Bartholomai JA, Balgemann RN, et al. Prediction of lung cancer patient survival via supervised machine learning classification techniques. Int J Med Inform. 2017;108:1–8. doi: 10.1016/j.ijmedinf.2017.09.013 29132615 PMC5726571

[11] Liu X, Hou Y, Wang X, Yu L, Wang X, Jiang L, et al. Machine learning-based development and validation of a scoring system for progression-free survival in liver cancer. Hepatol Int. 2020;14(4):567–576. doi: 10.1007/s12072-020-10046-w 32556865

[12] Kalafi EY, Nor NAM, Taib NA, Ganggayah MD, Town C, Dhillon SK. Machine Learning and Deep Learning Approaches in Breast Cancer Survival Prediction Using Clinical Data. Folia Biol (Praha). 2019;65(5–6):212–220. 32362304 10.14712/fb2019065050212

[13] Tahmassebi A, Wengert GJ, Helbich TH, Bago-Horvath Z, Alaei S, Bartsch R, et al. Impact of Machine Learning With Multiparametric Magnetic Resonance Imaging of the Breast for Early Prediction of Response to Neoadjuvant Chemotherapy and Survival Outcomes in Breast Cancer Patients. Invest Radiol. 2019;54(2):110–117. doi: 10.1097/RLI.0000000000000518 30358693 PMC6310100

[14] Poirion OB, Jing Z, Chaudhary K, Huang S, Garmire LX. DeepProg: an ensemble of deep-learning and machine-learning models for prognosis prediction using multi-omics data. Genome Med. 2021;13(1):112. doi: 10.1186/s13073-021-00930-x 34261540 PMC8281595

[15] Huang K, Zhang J, Yu Y, Lin Y, Song C. The impact of chemotherapy and survival prediction by machine learning in early Elderly Triple Negative Breast Cancer (eTNBC): a population based study from the SEER database. BMC Geriatr. 2022;22(1):268. doi: 10.1186/s12877-022-02936-5 35361134 PMC8973884

[16] Lou SJ, Hou MF, Chang HT, Lee HH, Chiu CC, Yeh SJ, et al. Breast Cancer Surgery 10-Year Survival Prediction by Machine Learning: A Large Prospective Cohort Study.Biology (Basel). 2021;11(1):47. doi: 10.3390/biology11010047 35053045 PMC8773427

[17] Jang BS, Kim IA. Machine-learning algorithms predict breast cancer patient survival from UK Biobank whole-exome sequencing data. Biomark Med. 2021;15(16):1529–1539. doi: 10.2217/bmm-2021-0280 34651513

[18] Tao W, Lu M, Zhou X, Montemezzi S, Bai G, Yue Y, et al. Machine Learning Based on Multi-Parametric MRI to Predict Risk of Breast Cancer. Front Oncol. 2021;11:570747. doi: 10.3389/fonc.2021.570747 33718131 PMC7952867

[19] Aamir S, Rahim A, Aamir Z, Abbasi SF, Khan MS, Alhaisoni M, et al. Predicting Breast Cancer Leveraging Supervised Machine Learning Techniques. Comput Math Methods Med. 2022;2022:5869529. doi: 10.1155/2022/5869529 36017156 PMC9398810

[20] Zhou CM, Xue Q, Wang Y, Tong J, Ji M, Yang JJ. Machine learning to predict the cancer-specific mortality of patients with primary non-metastatic invasive breast cancer. Surg Today. 2021;51(5):756–763. doi: 10.1007/s00595-020-02170-9 33104877

[21] Din NMU, Dar RA, Rasool M, Assad A. Breast cancer detection using deep learning: Datasets, methods, and challenges ahead. Comput Biol Med. 2022;149:106073. doi: 10.1016/j.compbiomed.2022.106073 36103745

[22] Radzi SFM, Karim MKA, Saripan MI, Rahman MAA, Isa INC, Ibahim MJ. Hyperparameter Tuning and Pipeline Optimization via Grid Search Method and Tree-Based AutoML in Breast Cancer Prediction. J Pers Med. 2021;11(10):978. doi: 10.3390/jpm11100978 34683118 PMC8540332

[23] Pfob A, Mehrara BJ, Nelson JA, Wilkins EG, Pusic AL, Sidey-Gibbons C. Towards Patient-Centered Decision-Making in Breast Cancer Surgery: Machine Learning to Predict Individual Patient-Reported Outcomes at 1-Year Follow-up. Ann Surg. 2021;277(1):e144–52. doi: 10.1097/SLA.0000000000004862 33914464 PMC9762704

[24] Xiao J, Mo M, Wang Z, Zhou C, Shen J, Yuan J, et al. The Application and Comparison of Machine Learning Models for the Prediction of Breast Cancer Prognosis: Retrospective Cohort Study. JMIR Med Inform. 2022;10(2):e33440. doi: 10.2196/33440 35179504 PMC8900909

[25] Eun NL, Kang D, Son EJ, Youk JH, Kim JA, Gweon HM. Texture analysis using machine learning-based 3-T magnetic resonance imaging for predicting recurrence in breast cancer patients treated with neoadjuvant chemotherapy. Eur Radiol. 2021;31(9):6916–6928. doi: 10.1007/s00330-021-07816-x 33693994

[26] Jiang X, Xu C. Deep Learning and Machine Learning with Grid Search to Predict Later Occurrence of Breast Cancer Metastasis Using Clinical Data. J Clin Med. 2022;11(19):5772. doi: 10.3390/jcm11195772 36233640 PMC9570670

[27] Liu P, Fu B, Yang SX, Deng L, Zhong X, Zheng H. Optimizing Survival Analysis of XGBoost for Ties to Predict Disease Progression of Breast Cancer. IEEE Trans Biomed Eng. 2021;68(1):148–160. doi: 10.1109/TBME.2020.2993278 32406821

[28] Rabinovici-Cohen S, Fernández XM, Grandal Rejo B, Hexter E, Hijano Cubelos O, Pajula J, et al. Multimodal Prediction of Five-Year Breast Cancer Recurrence in Women Who Receive Neoadjuvant Chemotherapy. Cancers (Basel). 2022;14(16):3848. doi: 10.3390/cancers14163848 36010844 PMC9405765

[29] Liu H, Kurc T. Deep learning for survival analysis in breast cancer with whole slide image data. Bioinformatics. 2022;38(14):3629–3637. doi: 10.1093/bioinformatics/btac381 35674341 PMC9272797

[30] Delen D, Walker G, Kadam A. Predicting breast cancer survivability: a comparison of three data mining methods. Artif Intell Med. 2005;34(2):113–27. doi: 10.1016/j.artmed.2004.07.002 15894176

[31] Surveillance, Epidemiology, and End Results Program [Internet]. Maryland: National Cancer Institute; c2022 [cited 2022 Oct 4]. SEER Program Overview; [about 2 screens]. Available from: https://seer.cancer.gov/about/overview.html

[32] Kursa MB, Rudnicki WR. Feature Selection with the Boruta Package. Journal of Statistical Software. 2010;36(11):1–13. 10.18637/jss.v036.i11

[33] Kuhn M. caret: Classification and Regression Training. Version 6.0–93 [R package]. 2022 Aug 9 [cited 2022 Oct 4]. Available from: https://CRAN.R-project.org/package=caret

[34] Chang W, Cheng J, Allaire JJ, Sievert C, Schloerke B, Xie Y, et al. shiny: Web Application Framework for R. Version 1.7.2 [R package]. 2022 Jul 19 [cited 2022 Oct 4]. Available from: https://CRAN.R-project.org/package=shiny

[35] Chang W, Ribeiro BB. shinydashboard: Create Dashboards with ’Shiny’. Version 0.7.2 [R package]. 2021 Sep 30 [cited 2022 Oct 4]. Available from: https://CRAN.R-project.org/package=shinydashboard

[36] Lorena AC, Jacintho LFO, Siqueira MF, Giovanni RD, Lohmann LG, de Carvalho ACPLF, et al. Comparing machine learning classifiers in potential distribution modelling. Expert Systems with Applications. 2011;38(5):5268–5275. doi: 10.1177/1747493018778713

[37] Meyer D, Dimitriadou E, Hornik K, Weingessel A, Leisch F. e1071: Misc Functions of the Department of Statistics, Probability Theory Group (Formerly: E1071), TU Wien. Version 1.7–11 [R package]. 2022 Jun 7 [cited 2022 Oct 4]. Available from: https://CRAN.R-project.org/package=e1071

[38] Grouven U, Bergel F, Schultz A. Implementation of linear and quadratic discriminant analysis incorporating costs of misclassification. Comput Methods Programs Biomed. 1996:49(1):55–60. doi: 10.1016/0169-2607(95)01705-4 8646839

[39] Venables WN, Ripley BD. Modern Applied Statistics with S. 4th ed. New York: Springer; 2002.

[40] Rashidi HH, Tran NK, Betts EV, Howell LP, Green R. Artificial Intelligence and Machine Learning in Pathology: The Present Landscape of Supervised Methods. Acad Pathol. 2019:6:2374289519873088. doi: 10.1177/2374289519873088 31523704 PMC6727099

[41] Schliep K, Hechenbichler K, Lizee A. kknn: Weighted k-Nearest Neighbors. Version 1.3.1 [R package]. 2016 Mar 26 [cited 2022 Oct 4]. Available from: https://CRAN.R-project.org/package=kknn

[42] Karatzoglou A, Smola A, Hornik K. kernlab: Kernel-Based Machine Learning Lab. Version 0.9–31 [R package]. 2022 Jun 9 [cited 2022 Oct 4]. Available from: https://CRAN.R-project.org/package=kernlab

[43] Bagante F, Spolverato G, Merath K, Weiss M, Alexandrescu S, Marques HP, et al. Intrahepatic cholangiocarcinoma tumor burden: A classification and regression tree model to define prognostic groups after resection. Surgery. 2019;166(6):983–990. doi: 10.1016/j.surg.2019.06.005 31326191

[44] Therneau T, Atkinson B. rpart: Recursive Partitioning and Regression Trees. Version 4.1.16 [R package]. 2022 Jan 24 [cited 2022 Oct 4]. Available from: https://CRAN.R-project.org/package=rpart

[45] Liaw A, Wiener M. Classification and Regression by randomForest. 2002;2(3):18–22.

[46] Vanegas J, Vásquez F. [Multivariate Adaptive Regression Splines (MARS), an alternative for the analysis of time series]. Gac Sanit. 2017:31(3):235–237. doi: 10.1016/j.gaceta.2016.10.003 28007311

[47] Friedman JH. Multivariate Adaptive Regression Splines. 1991;19(1):1–67. 10.1214/aos/1176347963

[48] Chen T, Guestrin C. XGBoost: A Scalable Tree Boosting System. Proceedings of the 22nd ACM SIGKDD InternationAl Conference on Knowledge Discovery and Data Mining; 2016 Aug 13–17; San Francisco, USA. 2016. p. 785–794.

[49] Huang YC, Li SJ, Chen M, Lee TS, Chien YN. Machine-Learning Techniques for Feature Selection and Prediction of Mortality in Elderly CABG Patients. Healthcare (Basel). 2021;9(5):547. doi: 10.3390/healthcare9050547 34067148 PMC8151160

[50] Chen T, He T, Benesty M, Khotilovich V, Tang Y, Cho H, et al. xgboost: Extreme Gradient Boosting. Version 1.6.0.1 [R package]. 2022 Apr 16 [cited 2022 Oct 4]. Available from: https://CRAN.R-project.org/package=xgboost

[51] Milborrow S. Notes on the earth package [Internet]. 2021 [cited 2022 Oct 4]. Available from: http://www.milbo.org/doc/earth-notes.pdf

[52] Stark GF, Hart GR, Nartowt BJ, Deng J. Predicting breast cancer risk using personal health data and machine learning models. PLoS One. 2019;14(12):e0226765. doi: 10.1371/journal.pone.0226765 31881042 PMC6934281

[53] Kantor O, Bao J, Jaskowiak N, Yao K, Tseng J. The Prognostic Value of the AJCC 8th Edition Staging System for Patients Undergoing Neoadjuvant Chemotherapy for Breast Cancer. Ann Surg Oncol. 2020;27(2):352–358. doi: 10.1245/s10434-019-07636-w 31376037

[54] Wang J, Lian CL, Zhou P, Lei J, Hua L, He ZY, et al. The prognostic and predictive value of the 8th American Joint Committee on Cancer (AJCC) staging system among early breast cancer patients aged <50 years. Gland Surg. 2021;10(1):233–241. doi: 10.21037/gs-20-587 33633979 PMC7882352

[55] Zhou J, Lei J, Wang J, Lian CL, Hua L, Yang LC, et al. Validation of the 8(th) edition of the American Joint Committee on Cancer Pathological Prognostic Staging for young breast cancer patients. Aging (Albany NY). 2020;12(8):7549–7560. doi: 10.18632/aging.103111 32320950 PMC7202534

[56] Shao N, Xie C, Shi Y, Ye R, Long J, Shi H, et al. Comparison of the 7th and 8th edition of American Joint Committee on Cancer (AJCC) staging systems for breast cancer patients: a Surveillance, Epidemiology and End Results (SEER) Analysis. Cancer Manag Res. 2019;11:1433–1442. doi: 10.2147/CMAR.S185212 30863154 PMC6388984

[57] Nindrea RD, Aryandono T, Lazuardi L, Dwiprahasto I. Diagnostic Accuracy of Different Machine Learning Algorithms for Breast Cancer Risk Calculation: a Meta-Analysis. Asian Pac J Cancer Prev. 2018;19(7):1747–1752. doi: 10.22034/APJCP.2018.19.7.1747 30049182 PMC6165638

[58] Chen MT, Sun HF, Zhao Y, Fu WY, Yang LP, Gao SP, et al. Comparison of patterns and prognosis among distant metastatic breast cancer patients by age groups: a SEER population-based analysis. Sci Rep. 2017;7(1):9254. doi: 10.1038/s41598-017-10166-8 28835702 PMC5569011

[59] Ji H, Ai N, Li Q, Zhang K, Di W. Clinical pathologies of breast cancer in the elderly and youths and their prognosis. Pak J Med Sci. 2014;30(3):535–8. doi: 10.12669/pjms.303.4929 24948974 PMC4048501

[60] Meshkat M, Baghestani AR, Zayeri F, Khayamzadeh M, Akbari ME. Survival Rate and Prognostic Factors among Iranian Breast Cancer Patients. Iran J Public Health. 2020;49(2):341–350. 32461942 PMC7231718

[61] Bertucci F, Ng CKY, Patsouris A, Droin N, Piscuoglio S, Carbuccia N, et al. Genomic characterization of metastatic breast cancers. Nature. 2019;569(7757):560–564. doi: 10.1038/s41586-019-1056-z 31118521

