# Supplementary material for: Evaluation of machine learning algorithms for the prognosis of breast cancer from the Surveillance, Epidemiology, and End Results database
Source: PLoS One. 2023 Jan 26;18(1):e0280340. doi: 10.1371/journal.pone.0280340 (PMC9879508; doi:10.1371/journal.pone.0280340)
Supplement: S2 Checklist — (DOCX) [file pone.0280340.s002.docx]

STROBE Statement—checklist of items that should be included in reports of observational studies

|  | Item No. | Recommendation | Page  No. | Relevant text from manuscript |
| --- | --- | --- | --- | --- |
| **Title and abstract** | 1 | (*a*) Indicate the study’s design with a commonly used term in the title or the abstract | 2 | cohort study |
|  |  | (*b*) Provide in the abstract an informative and balanced summary of what was done and what was found | 2-3 | Results and Conclusions |
| Introduction | | | |  |
| Background/rationale | 2 | Explain the scientific background and rationale for the investigation being reported | 4-5 | Many complex factors affected the prognosis of cancer patients, so survival prediction for cancer patients was a challenging task. In this context, modern oncology has witnessed the growing interest in digital technology, and the integration of digital technology and large medical data had brought new hope for personalized medicine. Since ML models were susceptible to factors such as data sources, input variables, and software, several articles using ML to predict the prognosis of BC patients were controversial. |
| Objectives | 3 | State specific objectives, including any prespecified hypotheses | 5 | This study was intended to establish a reliable data analysis model by comparing the performance of 10 common ML algorithms and the traditional AJCC staging system based on a national database, and used this model in Web application development to provide a good individualized prediction for others. |
| Methods | | | |  |
| Study design | 4 | Present key elements of study design early in the paper | 7 | Using DeLong test to compare the AUC values of different ML algorithms and 7th AJCC stage in the optimal test set, the best performing model was selected for web application development by shiny package and shinydashboard package. We utilized the accuracy, F1-score, sensitivity, specificity, and AUC to evaluate the performance of models for each prediction case. |
| Setting | 5 | Describe the setting, locations, and relevant dates, including periods of recruitment, exposure, follow-up, and data collection | 6 | The data of BC patients for this study was acquired from the SEER database, and it included 154014 patients based on the fact that year of diagnosis was from 2010 to 2014, primary tumor site was coded as C50.0 to C50.6 (including C50.0-Nipple, C50.1-Central portion of breast, C50.2-Upper-inner quadrant of breast, C50.3-Lower-inner quadrant of breast, C50.4-Upper-outer quadrant of breast, C50.5-Lower-outer quadrant of breast, C50.6-Axillary tail of breast), behavior recode for analysis was malignant, and diagnostic confirmation was positive histology. The study enrolled a total of 63145 patients by excluding patients with missing data and patients who survival time was less than 60 months and survival status was alive. The final endpoints of this study were the 5-year overall survival (OS) rate, so we excluded patients who survival time was less than 60 months and survival status was alive. |
| Participants | 6 | (*a*) *Cohort study*—Give the eligibility criteria, and the sources and methods of selection of participants. Describe methods of follow-up  *Case-control study*—Give the eligibility criteria, and the sources and methods of case ascertainment and control selection. Give the rationale for the choice of cases and controls  *Cross-sectional study*—Give the eligibility criteria, and the sources and methods of selection of participants | 6 | The data of BC patients for this study was acquired from the SEER database, and it included 154014 patients based on the fact that year of diagnosis was from 2010 to 2014, primary tumor site was coded as C50.0 to C50.6 (including C50.0-Nipple, C50.1-Central portion of breast, C50.2-Upper-inner quadrant of breast, C50.3-Lower-inner quadrant of breast, C50.4-Upper-outer quadrant of breast, C50.5-Lower-outer quadrant of breast, C50.6-Axillary tail of breast), behavior recode for analysis was malignant, and diagnostic confirmation was positive histology. The study enrolled a total of 63145 patients by excluding patients with missing data and patients who survival time was less than 60 months and survival status was alive. The final endpoints of this study were the 5-year overall survival (OS) rate, so we excluded patients who survival time was less than 60 months and survival status was alive. |
|  |  | (*b*) *Cohort study*—For matched studies, give matching criteria and number of exposed and unexposed  *Case-control study*—For matched studies, give matching criteria and the number of controls per case |  |  |
| Variables | 7 | Clearly define all outcomes, exposures, predictors, potential confounders, and effect modifiers. Give diagnostic criteria, if applicable | 6 | The data of BC patients for this study was acquired from the SEER database, and it included 154014 patients based on the fact that year of diagnosis was from 2010 to 2014, primary tumor site was coded as C50.0 to C50.6 (including C50.0-Nipple, C50.1-Central portion of breast, C50.2-Upper-inner quadrant of breast, C50.3-Lower-inner quadrant of breast, C50.4-Upper-outer quadrant of breast, C50.5-Lower-outer quadrant of breast, C50.6-Axillary tail of breast), behavior recode for analysis was malignant, and diagnostic confirmation was positive histology. The study enrolled a total of 63145 patients by excluding patients with missing data and patients who survival time was less than 60 months and survival status was alive (**Fig 1**). The final endpoints of this study were the 5-year overall survival (OS) rate, so we excluded patients who survival time was less than 60 months and survival status was alive. |
| Data sources/ measurement | 8* | For each variable of interest, give sources of data and details of methods of assessment (measurement). Describe comparability of assessment methods if there is more than one group | 7 | We used the Boruta package in the R software for feature selection and found that 14 attributes other than origin of primary were confirmed important. In order to reduce the over-fitting of the model and ensure the robustness of the model, we used the 9-fold cross-validation method to select the test set with the centered area under the curve (AUC) value as the optimal test set by the caret package. Using DeLong test to compare the AUC values of different ML algorithms and 7th AJCC stage in the optimal test set, the best performing model was selected for web application development by shiny package and shinydashboard package. We utilized the accuracy, F1-score, sensitivity, specificity, and AUC to evaluate the performance of models for each prediction case. |
| Bias | 9 | Describe any efforts to address potential sources of bias | 6 | The study enrolled a total of 63145 patients by excluding patients with missing data and patients who survival time was less than 60 months and survival status was alive. The final endpoints of this study were the 5-year overall survival (OS) rate, so we excluded patients who survival time was less than 60 months and survival status was alive. |
| Study size | 10 | Explain how the study size was arrived at | 6 | The data of BC patients for this study was acquired from the SEER database, and it included 154014 patients based on the fact that year of diagnosis was from 2010 to 2014, primary tumor site was coded as C50.0 to C50.6 (including C50.0-Nipple, C50.1-Central portion of breast, C50.2-Upper-inner quadrant of breast, C50.3-Lower-inner quadrant of breast, C50.4-Upper-outer quadrant of breast, C50.5-Lower-outer quadrant of breast, C50.6-Axillary tail of breast), behavior recode for analysis was malignant, and diagnostic confirmation was positive histology. The study enrolled a total of 63145 patients by excluding patients with missing data and patients who survival time was less than 60 months and survival status was alive. The final endpoints of this study were the 5-year overall survival (OS) rate, so we excluded patients who survival time was less than 60 months and survival status was alive. |

Continued on next page

| Quantitative variables | 11 | Explain how quantitative variables were handled in the analyses. If applicable, describe which groupings were chosen and why | 7 | We used the Boruta package in the R software for feature selection and found that 14 attributes other than origin of primary were confirmed important. Using DeLong test to compare the area under the curve (AUC) values of different ML algorithms and 7th AJCC stage in the test set, the best performing model was selected for web application development by shiny package and shinydashboard package. |
| --- | --- | --- | --- | --- |
| Statistical methods | 12 | (*a*) Describe all statistical methods, including those used to control for confounding | 7 | Using DeLong test to compare the area under the curve (AUC) values of different ML algorithms and 7th AJCC stage in the test set. |
|  |  | (*b*) Describe any methods used to examine subgroups and interactions | 7 | Statistical analysis were conducted using R software 4.1.0. |
|  |  | (*c*) Explain how missing data were addressed | 6 | The study enrolled a total of 63145 patients by excluding patients with missing data and patients who survival time was less than 60 months and survival status was alive. |
|  |  | (*d*) *Cohort study*—If applicable, explain how loss to follow-up was addressed  *Case-control study*—If applicable, explain how matching of cases and controls was addressed  *Cross-sectional study*—If applicable, describe analytical methods taking account of sampling strategy | 6 | The study enrolled a total of 63145 patients by excluding patients with missing data and patients who survival time was less than 60 months and survival status was alive. The final endpoints of this study were the 5-year overall survival (OS) rate, so we excluded patients who survival time was less than 60 months and survival status was alive. |
|  |  | (*e*) Describe any sensitivity analyses | 7 | In order to reduce the over-fitting of the model and ensure the robustness of the model, we used the 9-fold cross-validation method to select the test set with the centered area under the curve (AUC) value as the optimal test set by the caret package. |
| Results | | | | |
| Participants | 13* | (a) Report numbers of individuals at each stage of study—eg numbers potentially eligible, examined for eligibility, confirmed eligible, included in the study, completing follow-up, and analysed | - | No applicable |
|  |  | (b) Give reasons for non-participation at each stage | - | No applicable |
|  |  | (c) Consider use of a flow diagram | 6 | Fig 1. The inclusion and exclusion process of this study. |
| Descriptive data | 14* | (a) Give characteristics of study participants (eg demographic, clinical, social) and information on exposures and potential confounders | 11-12 | Table 1. Descriptive characteristics of 63145 BC patients. |
|  |  | (b) Indicate number of participants with missing data for each variable of interest | 11-12 | Table 1. Descriptive characteristics of 63145 BC patients. |
|  |  | (c) *Cohort study*—Summarise follow-up time (eg, average and total amount) | 11 | As of the follow-up time (November 2019), a total of 15734 patients died, and the 5-year OS was 75.1%. |
| Outcome data | 15* | *Cohort study*—Report numbers of outcome events or summary measures over time | 11 | As of the follow-up time (November 2019), a total of 15734 patients died, and the 5-year OS was 75.1%. |
|  |  | *Case-control study—*Report numbers in each exposure category, or summary measures of exposure |  |  |
|  |  | *Cross-sectional study—*Report numbers of outcome events or summary measures |  |  |
| Main results | 16 | (*a*) Give unadjusted estimates and, if applicable, confounder-adjusted estimates and their precision (eg, 95% confidence interval). Make clear which confounders were adjusted for and why they were included | 13 | Table 2. The accuracy, F1-score, sensitivity, specificity, and AUC value of 10 ML algorithms and 7th AJCC stage in the test set. |
|  |  | (*b*) Report category boundaries when continuous variables were categorized | 13 | Table 2. The accuracy, F1-score, sensitivity, specificity, and AUC value of 10 ML algorithms and 7th AJCC stage in the test set. |
|  |  | (*c*) If relevant, consider translating estimates of relative risk into absolute risk for a meaningful time period | Nothing | Nothing |

Continued on next page

| Other analyses | 17 | Report other analyses done—eg analyses of subgroups and interactions, and sensitivity analyses | nothing | nothing |
| --- | --- | --- | --- | --- |
| Discussion | | | | |
| Key results | 18 | Summarise key results with reference to study objectives | 19 | The comparative study of multiple forecasting models utilizing a large data noted that MARS based model achieved a much better performance compared to other ML algorithms and 7th AJCC stage in individualized estimation of survival of BC patients, which was very likely to be the next step towards precision medicine. |
| Limitations | 19 | Discuss limitations of the study, taking into account sources of potential bias or imprecision. Discuss both direction and magnitude of any potential bias | 18-19 | This study also had several limitations. Firstly, the SEER database lacked some data effected on the prognosis of patients, such as postoperative complications, surgical margin, and recurrence. Secondly, the models in this study were all trained and tested on different parts of the same data set. Ideally, the model would be trained on one data set and validated on another separately studied data set. This external verification could prove the universality of the model. We could not use another external data set for external verification, so we had to divide the data set into train set and test set. Although this research used the 9-fold cross-validation method to reduce the over-fitting of the model and ensure the robustness of the model, whether these ML models could be well generalized to new data sets required further research. Thirdly, compared with traditional statistical models, ML algorithms had black box characteristics. Interpretation and understanding of the ML model was a key issue. Fourthly, since there were various types of machine learning models, this study only compared 10 ML algorithms, which may affect the results of this study. Future work could be carried out to get a more accurate predictive model by including more ML algorithms. |
| Interpretation | 20 | Give a cautious overall interpretation of results considering objectives, limitations, multiplicity of analyses, results from similar studies, and other relevant evidence | 19 | The comparative study of multiple forecasting models utilizing a large data noted that MARS based model achieved a much better performance compared to other ML algorithms and 7th AJCC stage in individualized estimation of survival of BC patients, which was very likely to be the next step towards precision medicine. |
| Generalisability | 21 | Discuss the generalisability (external validity) of the study results | 18 | Nonetheless, there were some advantages of this research. Firstly, the data of this study came from the SEER database, which was one of the most representative large tumor databases in North America. Moreover, we compared the accuracy, F1-score, sensitivity, specificity, and AUC values of 10 ML algorithms in detail and reported the P values of the AUC values, while some other studies used less than 5 ML algorithms and rarely reported their P values [7-9,14]. More significantly, we used the selected ML model in Web application development to provide a good individualized prediction for others online. |
| Other information | |  | | |
| Funding | 22 | Give the source of funding and the role of the funders for the present study and, if applicable, for the original study on which the present article is based | Nothing | No Funding. |

*Give information separately for cases and controls in case-control studies and, if applicable, for exposed and unexposed groups in cohort and cross-sectional studies.

**Note:** An Explanation and Elaboration article discusses each checklist item and gives methodological background and published examples of transparent reporting. The STROBE checklist is best used in conjunction with this article (freely available on the Web sites of PLoS Medicine at http://www.plosmedicine.org/, Annals of Internal Medicine at http://www.annals.org/, and Epidemiology at http://www.epidem.com/). Information on the STROBE Initiative is available at www.strobe-statement.org.
